# Immunogenicity and Toxicity of Different Adjuvants Can Be Characterized by Profiling Lung Biomarker Genes After Nasal Immunization

**DOI:** 10.3389/fimmu.2020.02171

**Published:** 2020-09-11

**Authors:** Eita Sasaki, Hideki Asanuma, Haruka Momose, Keiko Furuhata, Takuo Mizukami, Isao Hamaguchi

**Affiliations:** ^1^Department of Safety Research on Blood and Biological Products, National Institute of Infectious Diseases, Tokyo, Japan; ^2^Influenza Virus Research Center, National Institute of Infectious Diseases, Tokyo, Japan

**Keywords:** adjuvant, nasal vaccine, type 1 interferon, genomics, biomarker, influenza

## Abstract

The efficacy of vaccine adjuvants depends on their ability to appropriately enhance the immunogenicity of vaccine antigens, which is often insufficient in non-adjuvanted vaccines. Genomic analyses of immune responses elicited by vaccine adjuvants provide information that is critical for the rational design of adjuvant vaccination strategies. In this study, biomarker genes from the genomic analyses of lungs after priming were used to predict the efficacy and toxicity of vaccine adjuvants. Based on the results, it was verified whether the efficacy and toxicity of the tested adjuvants could be predicted based on the biomarker gene profiles after priming. Various commercially available adjuvants were assessed by combining them with the split influenza vaccine and were subsequently administered in mice through nasal inoculation. The expression levels of lung biomarker genes within 24 h after priming were analyzed. Furthermore, we analyzed the antibody titer, cytotoxic T lymphocyte (CTL) induction, IgG1/IgG2a ratio, leukopenic toxicity, and cytotoxicity in mice vaccinated at similar doses. The association between the phenotypes and the changes in the expression levels of biomarker genes were analyzed. The ability of the adjuvants to induce the production of antigen-specific IgA could be assessed based on the levels of *Timp1* expression. Furthermore, the expression of this gene partially correlated with the levels of other damage-associated molecular patterns in bronchoalveolar lavage fluid. Additionally, the changes in the expression of proteasome- and transporter-related genes involved in major histocompatibility complex class 1 antigen presentation could be monitored to effectively assess the expansion of CTL by adjuvants. The monitoring of certain genes is necessary for the assessment of leukopenic toxicity and cytotoxicity of the tested adjuvant. These results indicate that the efficacy and toxicity of various adjuvants can be characterized by profiling lung biomarker genes after the first instance of immunization. This approach could make a significant contribution to the development of optimal selection and exploratory screening strategies for novel adjuvants.

## Introduction

Vaccines composed of purified antigens are often poorly immunogenic. The extent, strength, and duration of immune response to vaccines should be enhanced to ensure long-lived immune memory and protection. Innate immunity can be developed using adjuvants (1). Adjuvants are substances that can enhance the immune response to vaccine antigens. The choice of adjuvant drastically affects the nature and magnitude of the adaptive immune response to vaccine antigens, primarily by affecting innate immunity (2).

Profiling the mechanism of action of adjuvants facilitates the rational designing of vaccination strategies based on heterogeneous combinations of vaccine formulations for priming and boosting and the prediction of adjuvant efficacy for a particular vaccine antigen (3–8). For example, the influenza split vaccine (SV) consists of hemagglutinin (HA) and neuraminidase (NA) that are present in the outer shell of influenza virus particles; however, the immunogenicity elicited by these is insufficient for providing protection against infection (9, 10). SV is predominantly known to induce immunity via T helper (Th) 2 immune response (11, 12), and the effect of the addition of an adjuvant that enhances cellular immunity by inducing Th1 immune response has been verified. For example, R848 (resiquimod) and CpG K3 [K type CpG ODN (class B ODN)] induce Th1 immunity and enhance immunogenicity against SV vaccine antigen in mice (13–15).

Conversely, adjuvants such as aluminum salts (alum) that predominantly induce Th2 immune responses are not suitable as adjuvants for SV vaccine (11, 12), and it is also speculated that they may increase the risk of allergy induction owing to the excessive activation of Th2 immune response. In the past, whole-virion inactivated influenza vaccine (WPV) had been administered in humans (16). This vaccine can induce cytotoxic T lymphocyte (CTL) response and antibody production (11, 12, 17), which are sufficient for providing protection against infections. However, since several side effects, including fever, were observed, primarily in children (18), it is rarely used in current clinical practices. WPV-like immune activation can be partially achieved by adding a type 1 interferon (IFN)-inducing adjuvant such as R848 to SV (13–15). However, R848 is known to induce influenza-like symptoms characterized by fatigue, chills, and fever in humans (19–21). These symptoms are similar to the side effects observed in WPV vaccination (15). Similar patterns of toxicity were also observed in cases of poly I:C administration (22–25), which indicates that excessive IFN induction may lead to the development of side effects comparable to those observed in WPV vaccination. Therefore, during adjuvant selection, we must consider the toxicity as well as the protective immunity conferred. A wide variety of adjuvants are expected to be developed in future; therefore, the development of adjuvant profiling strategies is of importance in vaccine development.

Systems biology approaches for the assessment or prediction immune responses from gene expression data have been used to study animal and human responses to various vaccines (26–35). In an adjuvant study, microarrays were used to examine immune responses at an early stage in mice (33, 34). In addition, a system that uses a mathematical model has been developed to determine the possibility of predicting adaptive immunity (36, 37). Such methods accelerate the development of adjuvants and contribute to the discovery of safer and more effective adjuvants.

Systems biology approaches are multidisciplinary techniques that use computational analysis and mathematical modeling to analyze multiple data types associated with complex biological interactions. A systems biological approach was first applied to characterize the immune response elicited in humans vaccinated with the yellow fever vaccine YF-17D (35). More recently, adjuvanted and non-adjuvanted influenza vaccines have been characterized as well (33). The application of system biology techniques to the study of vaccine-mediated immunology is referred to as “systems vaccinology.”

We have previously reported that it is possible to profile the immunogenicity of inactivated influenza vaccines by analyzing the expression patterns of lung genes within 16 h of priming using genomic analyses (38–42). In addition, we constructed a novel safety evaluation system for an adjuvanted inactivated influenza vaccine using WPV as a toxicity control via mathematical analyses (43). It has also been reported that these evaluation methods can be applied in mouse (40) and rat (38, 39) models, as well as for the evaluation of nasal influenza vaccine (41). However, it was not clear whether the gene expression data reflected the vaccine-induced protective immunity and toxicity typified by antibody production, CTL induction, cytotoxicity, IFN induction, and Th1/Th2 immunity balance. If detailed information on the protective immunity and toxicity of vaccine adjuvants can be derived from gene expression data obtained within 16 h of priming, it may reduce the adjuvant development time.

In this study, we developed a method to construct a profile of the protective immunity (efficacy of a vaccine) and toxicity of adjuvants based on the expression profiles of biomarker genes at 16 h after priming (Table 1). We previously conducted a WPV immunogenicity biomarker search in animals using microarray technology (38). We identified gene expression clusters in the lungs of rats at 16 h after inoculation that were characteristic of WPV inoculation and different from the expression pattern obtained after inoculation with SV or saline (38). We also analyzed the same at other time points (24 and 48 h) and in other organs, such as spleen, blood, and liver; however, we did not identify characteristic gene expression clusters in these organs (38). Therefore, the 18 clustered biomarker genes expressed in the lungs were designated as immunogenicity indicators for influenza vaccines (Table 1) (38). The biomarker gene expression levels were altered by several adjuvants contained in SV (41–43). As the present study focuses on influenza vaccine, we used the biomarker gene set similar to those used in our previous studies (39–43). We previously performed a logistic regression analysis to estimate the similarity between the immunogenicity rate of a test vaccine to that of WPV using biomarker gene expression profile data (43). This method assesses the immunogenicity of the vaccine by assessing the similarity of the biomarker gene expression levels induced by the vaccine to those induced by WPV. Logistic regression analysis was performed for all biomarker genes to derive regression equations for each to calculate the similarity of the immunogenicity of a vaccine to that of WPV (43). Therefore, the similarity of the immunogenicity of a test product can be predicted using the regression equations and the actual biomarker expression levels (43). In the present study, this method was used for analyzing gene expression data. All tested adjuvants were combined with influenza vaccine antigens to form adjuvanted influenza vaccine. The mice were nasally inoculated with the tested commercially available adjuvants, and the production of antigen-specific antibodies (serum IgG and bronchoalveolar lavage fluid IgA), leukopenic toxicity, cytotoxicity, CTL activation, and Th1/Th2 immune balance (IgG1/IgG2a) were evaluated. We verified whether the vaccine-induced responses could be predicted based on the changes in biomarker gene expression observed at 16 h after priming. Based on the results, we verified whether the efficacy and toxicity of an adjuvant can be predicted using biomarker gene expression data.

**Table 1:** Biomarker genes for the evaluation of the safety of influenza vaccines (41–43).

**Symbol**	**Official full name**	**Accession**
*Cxcl11*	Chemokine (C-X-C motif) ligand 11	NM_019494
*Cxcl9*	Chemokine (C-X-C motif) ligand 9	NM_008599
*Zbp1*	Z-DNA binding protein 1	NM_021394
*Mx2*	MX dynamin-like GTPase 2	NM_013606
*Irf7*	Interferon regulatory factor 7	NM_016850
*Lgals9*	Lectin, galactoside-binding, soluble, 9	NM_010708
*Ifi47*	Interferon gamma inducible protein 47	NM_008330
*Tapbp*	TAP binding protein (tapasin)	NM_001025313
*Csf1*	Colony stimulating factor (macrophage)	NM_007778
*Timp1*	Tissue inhibitor of metalloproteinase 1	NM_001044384
*Trafd1*	TRAF type zinc finger domain containing 1	NM_001163470
*Lgals3bp*	Lectin, galactoside-binding, soluble, 3 binding protein	NM_011150
*Psmb9*	Proteasome (proteasome, macropain) subunit, beta type, 9	NM_013585
*C2*	Complement component 2	NM_013484
*Tap2*	Transporter 2, ATP-binding cassette, sub-family B (MDR/TAP)	XM_006525355
*Ifrd1*	Interferon-related developmental regulator 1	NM_013562
*Psme1*	Proteasome (proteasome, macropain) activator subunit 1	NM_011189
*Ngfr*	Nerve growth factor receptor	NM_033217

## Materials and Methods

### Animals and Ethics Statement

Six-to-eight-week-old female BALB/c mice (16–22 g) were obtained from SLC (Tokyo, Japan). The mice were housed in rooms maintained at 23 ± 1°C and at 50 ± 10% relative humidity under a 12 h light/12 h dark cycle. The mice were acclimated for at least 3 days before the experiments commenced. The animal experiments were performed according to the guidelines of the Institutional Animal Care and Use Committee of the National Institute of Infectious Diseases (NIID), Tokyo, Japan. The study was reviewed and approved by the Institutional Animal Care and Use Committee of NIID.

### Influenza Vaccines and Adjuvants

The influenza A virus (A/New Caledonia/20/99; H1N1) SV and WPV were generously provided by the Kitasato Institute (Tokyo, Japan). WPV (whole-virion preparation of an inactivated influenza virus comprising three different types of inactivated whole-virion components: A/Newcaledonia/20/99 (H_1_N_1_), A/Hiroshima/52/2005 (H_3_N_2_), and B/Malaysia/2506/2004) used for leukopenic toxicity test were obtained from KM Biologics Co., Ltd. (Kumamoto, Japan). The following commercially available adjuvants were used: Aluminum hydroxide gel (Alhydrogel, alum), squalene-based oil-in-water adjuvant AddaVax, murine stimulator of interferon genes (STING) ligand DMXaa, toll-like receptor 1/2 (TLR1/2) agonist Pam3CSK4, TLR3 agonist Poly I:C, and TLR7/8 agonist R848 obtained from InvivoGen (San Diego, CA, USA). Silicon dioxide nanopowder (NanoSiO_2_, particle size 10–20 nm) was obtained from Sigma-Aldrich (MA, USA). The TLR9 agonist CpG K3 was generously provided by Prof. Ken J Ishii (The Institute of Medical Science, The University of Tokyo, Tokyo, Japan).

The dose and inoculation volume of influenza vaccine antigens and adjuvants are outlined in Table 2. The appropriate quantity of each adjuvant was mixed with SV and the final volume was unified in each experiment, as described in Table 2. Intranasal inoculation was performed after anesthetization by intraperitoneal injection of sodium phenobarbital.

**Table 2:** References for the dose, volume, and biomarker gene expression data of adjuvants tested for nasal vaccination (barring studies on leukopenic toxicity).

**Adjuvant**	**Adjuvant content (/mouse)**	**Antigen/Formulation**	**Dosing volume (/mouse)**	**References for biomarker gene expression data in Figure 1A**
Non	Non	A/New Caledonia/20/99; H1N1/Influenza split-product vaccine 1 μg/mouse	30 μL	Present study
Poly I:C	1, 5, or 10 μg	A/New Caledonia/20/99; H1N1/Influenza split-product vaccine 1 μg/mouse	30 μL	(43)
CpG K3	2, 5, or 10 μg	A/New Caledonia/20/99; H1N1/Influenza split-product vaccine 1 μg/mouse	30 μL	Present study
Aluminum	3, 10, 30, or 100 μg	A/New Caledonia/20/99; H1N1/Influenza split-product vaccine 1 μg/mouse	30 μL	(44)
AddaVax	12.5, 25, or 50% (v/v)	A/New Caledonia/20/99; H1N1/Influenza split-product vaccine 1 μg/mouse	30 μL	(43)
DMXaa	10, 30, 100, or 300 μg	A/New Caledonia/20/99; H1N1/Influenza split-product vaccine 1 μg/mouse	30 μL	(44)
NanoSiO_2_	3, 10, 30, or 100 μg	A/New Caledonia/20/99; H1N1/Influenza split-product vaccine 1 μg/mouse	30 μL	(44)
Pam3CSK4	3, 10, 30, or 100 μg	A/New Caledonia/20/99; H1N1/Influenza split-product vaccine 1 μg/mouse	30 μL	(44)
R848	2, 10, 25, or 50 μg	A/New Caledonia/20/99; H1N1/Influenza split-product vaccine 1 μg/mouse	30 μL	Present study
WPV	None	A/New Caledonia/20/99; H1N1/Influenza split-product vaccine 1 μg/mouse	30 μL	Present study

### Lung Biomarker Gene Expression Analyses

Data on biomarker gene expression levels in the lungs of Poly I:C-, alum-, AddaVax-, DMXaa-, NanoSiO_2_-, or Pam3CSK4-treated animals were obtained from the experimental data in our previous studies (43, 44). The details are outlined in Table 2. For the gene expression analyses of animals treated with other influenza vaccines and adjuvanted influenza vaccines, the biomarker gene expressions levels were analyzed based on methods reported earlier (40–44). Briefly, the lung lysates were prepared and the QuantiGene Plex (QGP) assay was performed, as described in our previous studies (40–44). The lung specimens were immediately stored in RNAlater (Thermo Fisher Scientific Japan, Kanagawa, Japan) and homogenized before the QGP assay was performed according to the instructions provided with the QuantiGene Plex Reagent System (Panomics/Affymetrix, Fremont, CA, USA), as described previously (40, 41). The probes for the biomarker genes were designed as described previously (41) (Table 1). WPV-like toxicity levels were calculated from the gene expression levels using ordinal logistic regression analysis with JMP 12.01 statistical software (SAS Institute, NC, USA), as previously reported (43). The biomarker genes were categorized according to the conventions for the Kyoto Encyclopedia of Genes and Genomes (KEGG) pathways using the GENECODIS program (https://genecodis.genyo.es) (Table 3).

**Table 3:** Pathway and function of the biomarker genes.

**Symbol**	**Gene name**	**Pathway and function (KEGG Pathway)**
*Mx2*	Mx dynamin-like GTPase 2	mmu05164:Influenza A; mmu05162:Measles
*Tapbp*	TAP binding protein	mmu04612; Antigen processing and presentation
*Zbp1*	Z-DNA binding protein 1	mmu04623:Cytosolic DNA-sensing pathway
*Cxcl11*	Chemokine (C-X-C motif) ligand 11	mmu04060:Cytokine-cytokine receptor interaction, mmu04062:Chemokine signaling pathway, mmu04620:Toll-like receptor signaling pathway
*Cxcl9*	Chemokine (C-X-C motif) ligand 9	mmu04060:Cytokine-cytokine receptor interaction, mmu04062:Chemokine signaling pathway, mmu04620:Toll-like receptor signaling pathway
*Csf1*	Colony stimulating factor 1 (macrophage)	mmu04014:Ras signaling pathway, mmu04015:Rap1 signaling pathway, mmu04060:Cytokine-cytokine receptor interaction, mmu04151:PI3KAkt signaling pathway, mmu04380:Osteoclast differentiation, mmu04640:Hematopoietic cell lineage, mmu04668:TNF signaling pathway, mmu05323:Rheumatoid arthritis
*C2*	Component 2	mmu04610:Complement and coagulation cascades, mmu05133:Pertussis, mmu05150:Staphylococcus aureus infection, mmu05322:Systemic lupus erythematosus,
*Ifi47*	Interferon gamma inducible protein 47	mmu04668:TNF signaling pathway
*Irf7*	Interferon regulatory factor 7	mmu04620:Toll-like receptor signaling pathway, mmu04622:RIG-I-like receptor signaling pathway, mmu04623:Cytosolic DNA-sensing pathway, mmu05160:Hepatitis C, mmu05161:Hepatitis B, mmu05162:Measles, mmu05164:Influenza A, mmu05168:Herpes simplex infection, mmu05203:Viral carcinogenesis
*Ngfr*	Ngfr nerve growth factor receptor (TNFR superfamily, member)	mmu04014:Ras signaling pathway, mmu04015:Rap1 signaling pathway, mmu04060:Cytokine-cytokine receptor interaction, mmu04151:PI3KAkt signaling pathway, mmu04722:Neurotrophin signaling pathway, mmu05202:Transcriptional misregulation in cancer
*Psme1*	Proteasome (prosome, macropain) activator subunit 1	mmu03050:Proteasome, mmu04612:Antigen processing and presentation
*Psmb9*	Proteasome (prosome, macropain) subunit, beta type 9	mmu03050:Proteasome
*Timp1*	Tissue inhibitor of metalloproteinase 1	mmu04066:HIF-1 signaling pathway
*Tap2*	Transporter 2, ATP binding cassette, subfamily B	mmu02010:ABC transporters, mmu04145:Phagosome, mmu04612:Antigen processing and presentation, mmu05168:Herpes simplex infection, mmu05340:Primary immunodeficiency

### Measurement of White Blood Cell (WBC) Counts and Serum IFN-α Concentration

To assess leukopenic toxicity, the leukopenic toxicity test was performed according to a method reported previously (42). The concentrations of the tested vaccine antigens (SV or WPV) were adjusted to 15 μg HA/0.5 mL. Each adjuvant was mixed with SV. The tested vaccine was injected intraperitoneally at a dose of 0.5 mL/mouse. At 16 h after injection, the mice were anesthetized using sodium pentobarbital. Blood samples were collected via the inferior vena cava. The WBC and platelet counts were determined using an automatic hemocytometer, the Celltac MEK-6450 (Nihon Kohden, Tokyo, Japan). Sera were isolated using Capiject (Terumo Medical Corporation, Somerset, NJ). The concentration of IFN-α in mice sera was measured using the Mouse IFN Alpha enzyme-linked immuno-sorbent assay (ELISA) Kit (PBL InterferonSource, Piscataway, NJ, USA).

### Collection of Bronchoalveolar Lavage Fluid (BALF) and Measurement of Double-Stranded DNA (dsDNA) Concentration in BALF

The mice were sacrificed by an overdose of sodium pentobarbital. After cannulating the trachea, the lungs were lavaged with 1% BSA containing PBS (1.0 mL). The supernatants of the BALF samples were collected after centrifugation at 3,000 × g at 4°C for 15 min. The concentrations of interleukin (IL)-33 and IL-1α in BALF were measured using cytokine ELISA kits (R&D Systems, Minneapolis, MN, USA) according to the manufacturer's instructions. According to the methods described in a previous study (45), the concentration of dsDNA in BALF was measured using the Quant-iTTM Picogreen® dsDNA Assay Kit (Invitrogen, San Diego, CA, USA).

### Measurement of Antigen-Specific Antibody Concentration

Mice were nasally immunized with influenza SV plus each adjuvant or WPV twice at a 4-week interval. The vaccine doses and the quantity of adjuvant and antigens administered are outlined in Table 2. Two weeks after the final immunization, the mice were sacrificed by an overdose of sodium pentobarbital. Nasal washes, BALF, and sera were collected 2 weeks after the final immunization, as described previously (46, 47). Each sample was centrifuged to remove cellular debris and subjected to HA-specific ELISA (48). The isotypes of HA-specific monoclonal antibodies in the nasal wash, BALF, and sera samples were determined using ELISA, as previously described (49, 50). Briefly, 96-well-Falcon microtest assay plates (BD Biosciences, San Jose, CA, USA) were coated with recombinant influenza A H_1_N_1_ (A/Puerto Rico/8/34) (PR8) HA protein or purified A/New Caledonia/20/99; H_1_N_1_ HA (1.0 μg/mL). After blocking (using 1% BSA in PBS), 2-fold serial dilutions of the samples were added and incubated overnight at 4°C. Horseradish peroxidase (HRP)-labeled goat anti-mouse μ, γ, or α heavy chain-specific antibodies (Abs) (Southern Biotechnology Associates, Birmingham, AL) were added, and the color was allowed to develop for 15 min at room temperature in 100 μL of 1.1 mM 2,2′-azino bis (3-ethylbenz-thiazoline-6-sulfonic acid) (EMD Biosciences, La Jolla, CA, USA). The antibody concentration was calculated based on a standard curve plotted using data obtained from experiments with commercially available mouse anti-PR8 monoclonal antibody and recombinant PR8 protein-coated 96-well-plates. Briefly, the sample was serially diluted, and the antibody concentration was determined from the standard curve from a range within which absorbance and dilution concentration followed a linear trend (*r*^2^ > 0.95).

### *In vivo* CTL Assay

Mice were nasally immunized with influenza SV plus adjuvant or WPV twice at a 4-week interval. One week after the final immunization, the immunized mice were used for *in vivo* CTL assay. The *in vivo* CTL assay was performed according to previous reports (51–53) with slight modifications. Briefly, spleen cells (1 × 10^7^ cells/mL) from naïve BALB/c mice were treated with 1 μM influenza virus HA peptide (IYSTVASSL)- or OVA (257–264)-peptide-containing PBS for 2 h at 37°C. After washing twice with PBS, the recovered cells (1 × 10^7^ cells/mL) were labeled with different concentrations of carboxyfluorescein diacetate succinimidyl ester (CFSE) (0.25 or 2.5 μM, Molecular Probes, Eugene, OR) at room temperature for 10 min. The labeling experiment was terminated by the addition of one-half volume of fetal calf serum followed by two additional washing steps. Five million cells carrying each peptide were mixed and injected intravenously (*i.v*.) into the immunized mice. At 14 h after injection, the spleens were harvested to prepare single-cell-suspensions, and the CFSE-positive cells were analyzed by flow cytometry with exclusion of dead cells by propidium iodide (BD Biosciences, CA, USA) staining. Nucleoprotein-specific killing was calculated as follows: specific killing (%) = {1 – [(number of cells carrying HA (533–541) in immunized mice (CFSE high))/(number of cells carrying OVA257–264 in immunized mice (CFSE low))]/[(number of cells carrying HA (533–541) in normal mice (CFSE high))/(number of cells carrying OVA257–264 in normal mice (CFSE low))]} × 100.

### Flow Cytometry Analysis of Antigen-Specific CTLs in Lungs

Two weeks after the second vaccination, single-cell suspensions of lung cells were washed and stained with the fluorescently labeled monoclonal antibody (mAb) fluorescein isothiocyanate-conjugated CD8a (clone 53-6.7, eBioscience) and a phycoerythrin-conjugated H2-K^d^ tetramer bearing the influenza HA peptide IYSTVASSL epitope (533–541) derived from Influenza A/Puerto Rico/8/34 (PR8, H_1_N_1_) (MBL International, MA, USA). Single-cell suspensions prepared using lung tissues were incubated with the tetramer for 60 min on ice in dark and with antibodies for 30 min on ice in dark. To exclude the dead cells from the analysis, the cells were stained with propidium iodide (BD Biosciences). Following incubation with the tetramers and mAbs for 30 min at 4°C in dark, the cells were washed twice and analyzed using a CytoFLEX Flow Cytometer (Beckman Coulter Inc., CA, USA). The acquired data were analyzed using FlowJo software (TreeStar, San Carlos, CA, USA).

### Statistical Analyses

Statistical analyses were performed with GraphPad Prism 6 (GraphPad Software, La Jolla, CA, USA). For multiple comparisons, one-way analysis of variance followed by Dunnett's multiple comparison test were performed. For the comparison of two groups, unpaired Student's *t*-test was performed. *P* < 0.05 was considered significant.

## Results

### Evaluation of Biomarker Gene Expression Levels From Genomic Analyses for the Prediction of WPV-Like Immunogenicity and Th1 Immunity Induced by Influenza Vaccine

**Figure 1:**
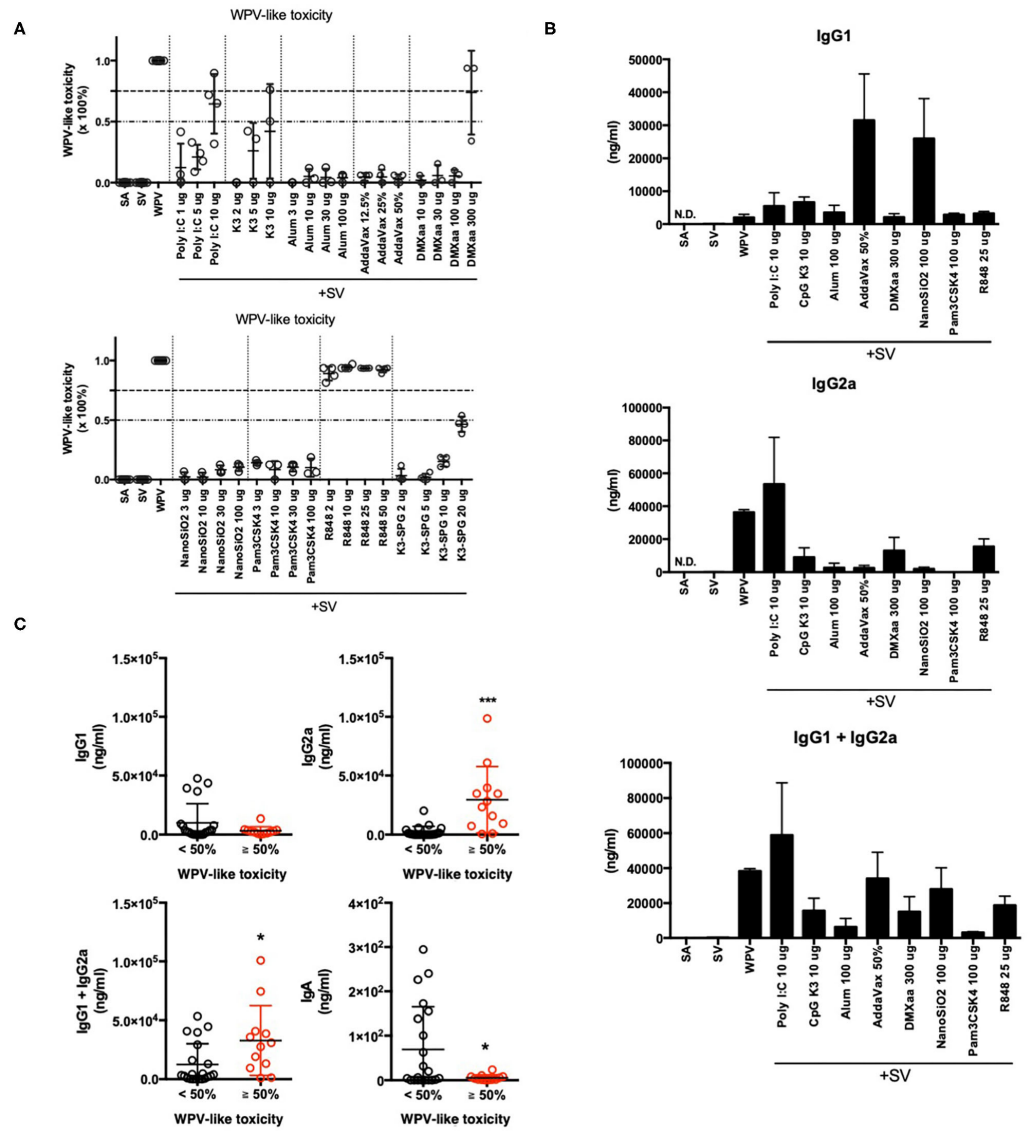
Total elevation in lung biomarker gene expression levels reflected WPV-like toxicity and IgG2a productivity. **(A,B)** Total elevation of lung biomarker gene expression levels at 16 h after intranasal priming with each commercially available adjuvant plus SV or WPV **(A)**, and serum antigen-specific IgG1 and IgG2a levels at 14 days after final vaccination **(B)**. The antigen-specific serum IgG levels were measured using ELISA. **(C)** Adjuvanted vaccine- or WPV-induced serum IgG and BALF IgA levels are indicated individually according to the induction of total lung biomarker gene expression, <50% or ≥ 50%. Results are expressed in terms of mean ± SEM [*n* = 3–5 for **(A,B)** or 12–25 for **(C)** in each group]. *p < 0.05 and ***p < 0.001 (Student's *t*-test).

We previously demonstrated that adjuvant safety in intranasal influenza vaccines can be assessed using biomarker genes sets (Table 1) (41, 43). In this model, the safety level was assessed using WPV as a toxicity reference (41, 43). Therefore, the safety scale has been referred to as WPV-like toxicity level in the present study. The results revealed the similarity of gene expression levels with those corresponding to WPV. This method is considered suitable for the evaluation of toxicity associated with IFN induction, as observed in cases of WPV treatment. In this study, we evaluated the WPV-like toxicity level of commercially available adjuvants-containing SV that were administered via nasal inoculation in mice. The results indicate that the WPV-like toxicity levels were higher in case of poly I:C-, CpG K3-, DMXaa-, and R848-adjuvanted SV (Figure 1A). These adjuvants have been known to induce the expression of type 1 IFN (54–57), which suggests that these adjuvants can induce immunogenicity and toxicity related to type 1 IFN. Among these, R848 and poly I:C have been known to cause side effects in humans, as observed in treatment with WPV (19–25). This result indicates that biomarker gene-based safety evaluation focused on WPV-like toxicity is useful for assessing the toxicity of adjuvants. The raw data for biomarker gene expression levels indicated in Figure 1 are outlined in Supplementary Table 1.

Type 1 IFN has been reported to contribute to vaccine efficacy by inducing antigen-specific antibody production (14) and CTL activation (51). The biomarker genes in lungs include several type 1 IFN-related genes (Table 1). To determine whether the elevation of biomarker gene expression levels is associated with vaccine antigen-specific antibody production, the levels of serum IgG1 (Th2 immunity), IgG2a (Th1 immunity), and BALF IgA were measured after nasal immunization was conducted twice (Figure 1B). Different elevation patterns were observed in the levels of serum IgG1 and IgG2a when different adjuvants were used (Figure 1B). Treatment with type 1 IFN-inducing adjuvants and vaccine, such as WPV, poly I:C, DMXaa, CpG K3, and R848, resulted in high serum IgG2a levels, whereas treatment with alum, AddaVax, and NanoSiO_2_, which elicit weak cytotoxicity (58–61), resulted in high serum IgG1 levels. To assess whether the WPV-like toxicity levels determined using biomarker gene analyses are suitable as IgG1, IgG2a, and IgA expression indicators, the WPV-like toxicity levels of each of adjuvant tested in Figure 1A were divided into two groups based on levels “< 50%” and “≥ 50%.” Next, the levels of antibody production in each group were analyzed individually (Figure 1C). The results indicated that the serum IgG1 levels did not differ significantly between the two groups. However, the serum IgG2a levels were significantly higher in the “≥ 50%” group (Figure 1C). Although the total IgG (IgG1 plus IgG2a) and BALF IgA levels between the two groups were considerably different, the difference was not significant. In the graph for BALF IgA, the dots indicating relatively high levels of IgA in the “< 50%” group correspond to AddaVax- and NanoSiO_2_-adjuvanted SV-immunized mice (Figure 1C). Therefore, WPV-like toxicity level prediction-based biomarker gene expression patterns could be useful for assessing the potential for inducing Th1 immunity using adjuvants, as indicated by the IgG2a levels in mice.

### Expression Profile of Different Biomarker Genes After Treatment With Adjuvanted Vaccines

**Figure 2:**
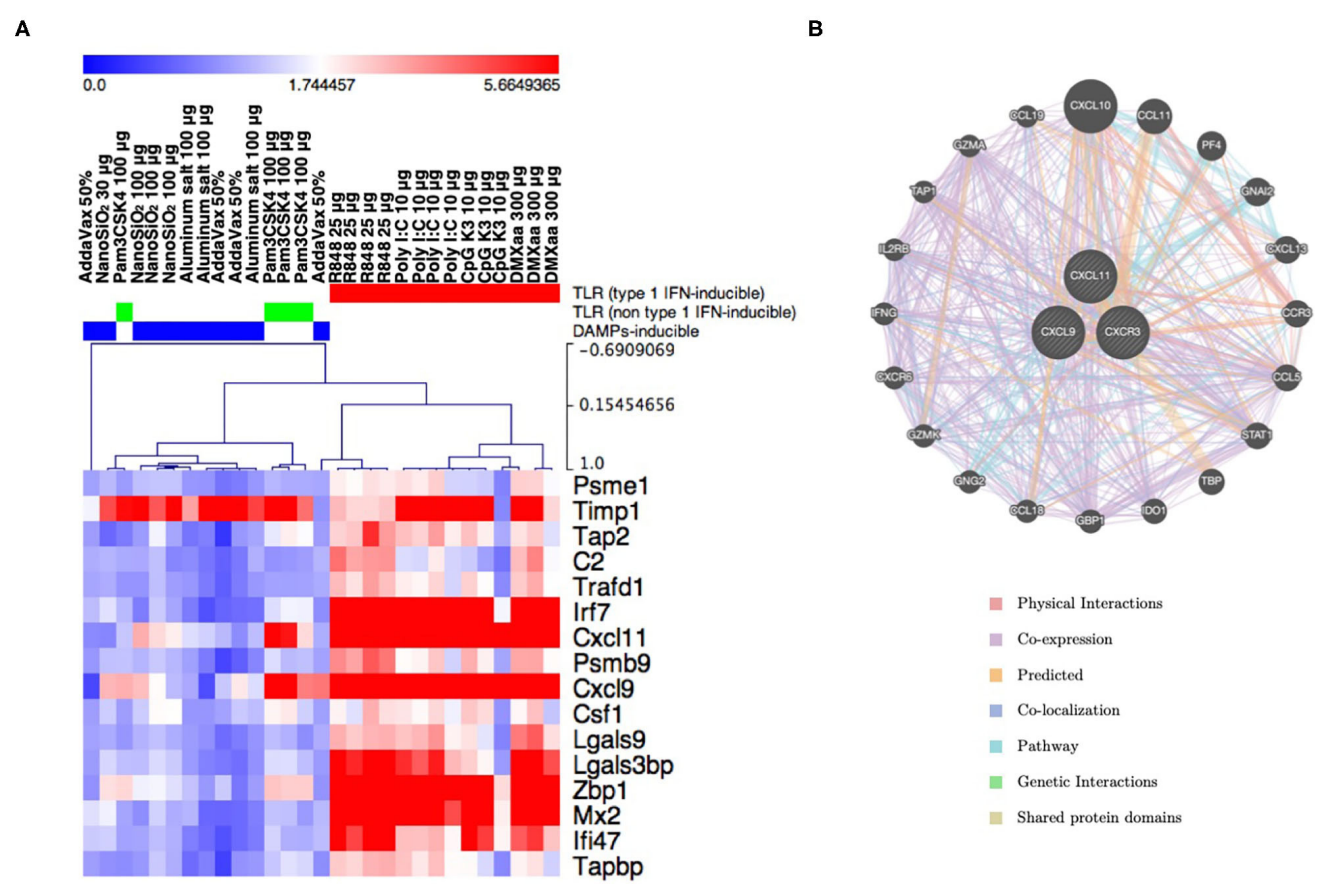
The expression pattern of individual lung biomarker genes characterizes the innate immunity induced by adjuvants. **(A)** Result of hierarchical clustering in lung biomarker genes and tested adjuvants. The clusters (TLR-related/type 1 IFN-inducing, TLR-related/non-type 1 IFN-inducing, and DAMP-inducing) are indicated by color bars at the top of the heat map. **(B)** Gene–gene interaction network constructed using GeneMANIA program. CXCL9 and CXCL11, which are included in the lung biomarker gene sets and influence the construction of the Pam3CSK4 cluster, along with their receptor CXCR3, are closely associated with genes related to Th1-, IFNγ-, and CTL-mediated immunity.

The safety evaluation system assessed the safety based on the average expression levels of biomarker genes (Figure 1A). This system did not indicate the characteristic expression fluctuation in each gene, as this could be masked when the values are averaged. Hierarchical clustering analysis was performed using the data for biomarker gene expression corresponding to each adjuvant tested (Figure 2A). The results indicated that the tested adjuvants could be primarily categorized in three groups based on biomarker expression patterns (Figure 2A), namely, type 1 IFN-inducing adjuvants (R848, poly I:C, CpG K3, and DMXaa), IFN-γ-inducing adjuvant (Pam3CSK4), and non-IFN-inducing adjuvants (AddaVax, alum, and NanoSiO_2_). In the type 1 IFN-inducing adjuvant group, the expression levels of type 1 IFN-related and -inducible genes, such as *Irf7, Zbp1, Mx2*, and *Cxcl9*, were notably elevated. However, the expression levels of the genes in the non-IFN-inducing adjuvant group, except that of *Timp1*, did not undergo notable elevation. Only the expression levels of *Cxcl11, Cxcl9*, and *Timp1* in the IFN-γ-inducible group underwent characteristic elevation (Figure 2A). We attempted to identify the genes likely to share function with these three genes based on their interactions using GeneMANIA (a gene–gene interaction network was automatically constructed and visualized using Cytoscape). *CXCL11, CXCL9*, and their receptor *CXCR3* form a network of Th1 immunity-related genes represented by *IFN-*γ, *STAT1, GZMK*, and *GZMA* (Figure 2B). Indeed, Pam3CSK4 has been reported to induce IFN-γ-mediated Th1 immunity (62). In our attempt to determine the function of each biomarker gene using the KEGG pathway, we observed that several biomarker genes assessed in this study were associated with antiviral response, cytokine response, TLR-mediated signaling, and antigen processing (Table 3, Supplementary Figure 1). These results indicate that biomarker gene expression profiling may serve as an effective tool for the prediction of the mode of action of vaccine adjuvants.

### Assessment of Type 1 IFN-Induced Leukopenic Toxicity Using the Three Biomarker Genes

**Figure 3:**
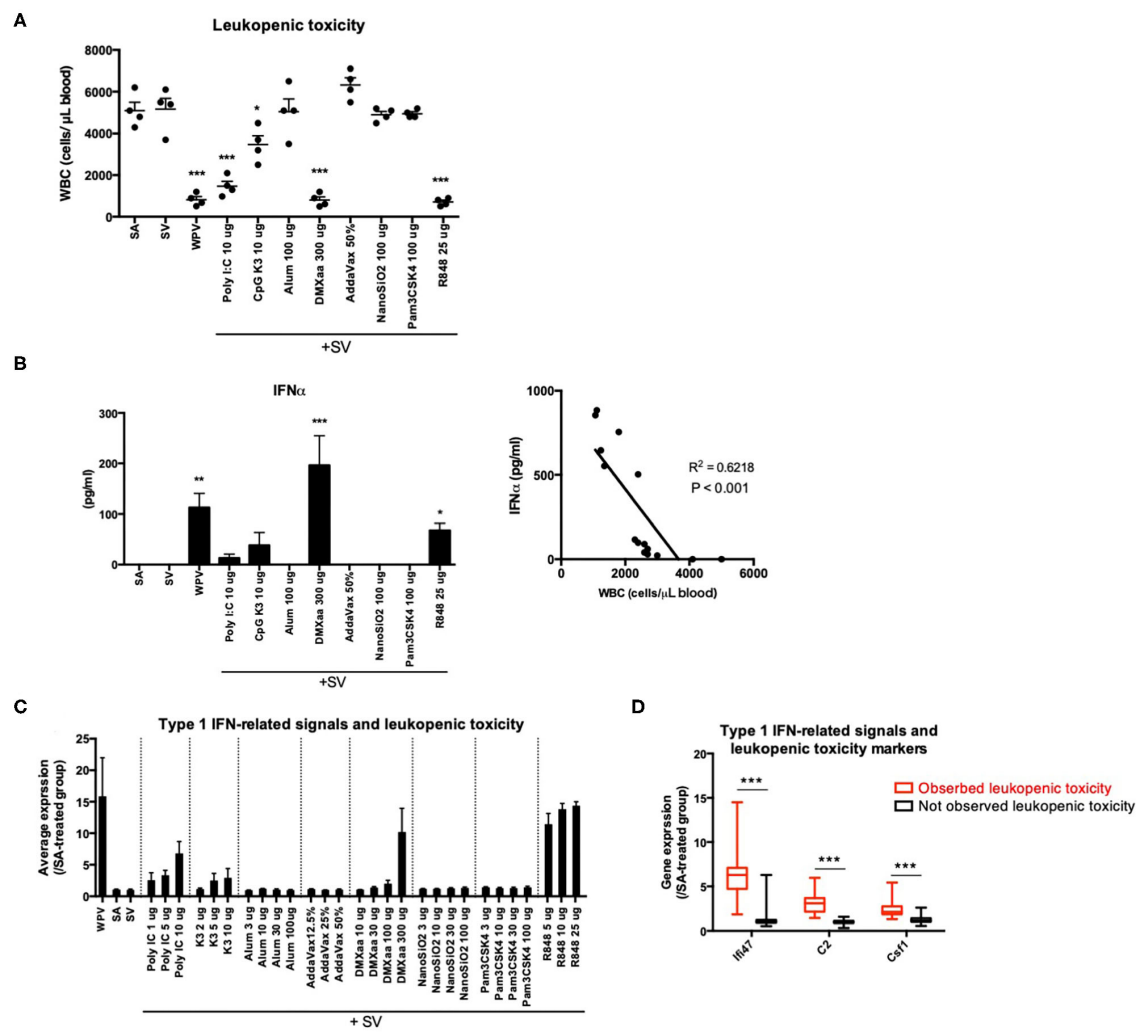
The expression levels of the three genes reflect WPV- or adjuvanted SV-induced leukopenic toxicity. Treatment with WPV or certain adjuvants plus SV induced leukopenic toxicity **(A)** accompanied with elevation in serum IFNα levels **(B)**. The tested vaccines were intraperitoneally injected and white blood cell counts were determined at 16 after injection according to the leukopenic toxicity test method described in the Materials and Methods. The changes in the average expression levels of the three genes after treatment with each vaccine plus adjuvants **(C)** and individual expression changes induced by the tested adjuvants are indicated individually according to the leukopenic toxicity **(D)**. Data are represented in box-and-whisker plots, which indicate the median value (black bar inside box), the 25th and 75th percentiles (bottom and top of box, respectively), and minimum and maximum values (bottom and top whisker, respectively) **(D)**. Other results are expressed in terms of mean ± SEM [*n* = 4 for **(A,B)**, 3–5 for **(C)**, or 37–100 for **(D)** in each group). *p < 0.05, **p < 0.01 and ***p < 0.001 compared with SA **(A,B)** (Dunnett's multiple comparison test). ***p < 0.001 **(D)** (Student's *t*-test).

Based on the results of KEGG pathway (Table 3) and GeneMANIA (Figure 2B) analyses, we suggested that the expression levels of individual genes can be assessed to predict the efficacy and toxicity of adjuvants. We examined whether the efficacy and toxicity levels of adjuvants can be distinguished based on the characteristic expression changes for each gene. First, adjuvant-induced leukopenic toxicity was demonstrated. We previously demonstrated that biomarker genes can be used as a tool for the assessment of leukopenic toxicity induced upon treatment with WPV (42). Leukopenic toxicity caused by influenza vaccination is considered to involve the excessive production of type 1 IFN (63). In addition to WPV, poly I:C has been reported to induce leukopenic toxicity in rabbit and mice (64, 65). In this study, the leukopenic toxicity induced by each adjuvanted vaccine that has the ability to induce type 1 IFN production (Figure 3A) was predicted. The rates of WBC reduction tended to correlate with the elevation in serum IFN-α concentration (Figure 3B). Among the lung biomarker genes, *Ifi47, C2*, and *Csf1*, which are useful genes for the assessment of WPV-induced leukopenic toxicity, were investigated in our previous study (42). The tested adjuvants were divided into two groups as follows: those that significantly reduced WBC levels compared to that in the SA group (eliciting leukopenic toxicity) and those that did not. The results indicated that the levels of the three biomarker genes were significantly elevated in the groups that exhibited leukopenic toxicity (Figures 3C,D). Therefore, the expression levels of each of *Ifi47, C2*, and *Csf1* at 16 h post priming can be used to assess leukopenic toxicity induced by these adjuvants.

### Increase in the Ratio of *Csf1* Plus *Timp1* Expression Levels to the Sum of Expression Levels of All Biomarker Genes as an Indicator of Potential for Th2 Immunity Activation

**Figure 4:**
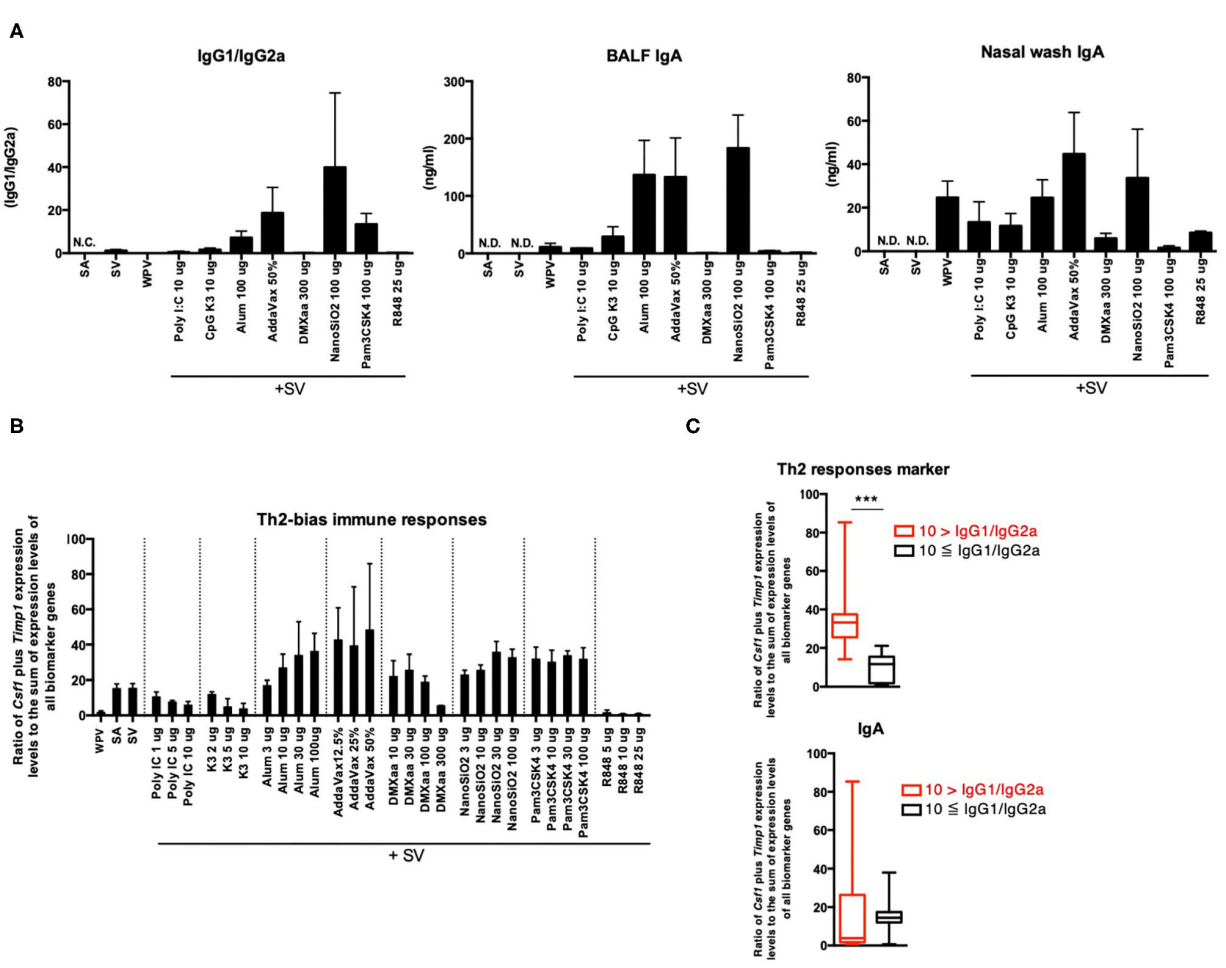
Ratio of *Csf1* plus *Timp1* expression levels to the sum of expression levels of all biomarker genes in lungs reflects Th2 immunity induced by adjuvants. **(A)** Antigen-specific serum IgG levels and BALF or nasal wash IgA levels 14 days after final vaccination. The changes in the ratio of *Csf1* plus *Timp1* expression levels to the sum of expression levels of all biomarker genes induced by the tested adjuvant **(B)** and in their levels were individually indicated according to the IgG1/IgG2a ratio **(C)**. Data are represented in box-and-whisker plots, which indicate the median value (black bar inside box), the 25th and 75th percentiles (bottom and top of box, respectively), and minimum and maximum values (bottom and top whisker, respectively) **(C)**. Other results are expressed in terms of mean ± SEM [*n* = 4 for **(A)**, 3–5 for **(B)**, or 16–53 for **(C)** in each group]. ***p < 0.001 (Student's *t*-test).

The balance between Th1 and Th2 immune responses is a critical determinant of vaccine efficacy. For example, SV predominantly induces Th2 immunity (11, 12); therefore, its potential for inducing Th1 immunity and CTL activation is low. An adjuvant that induces Th1 immunity would potentiate the immunogenicity of SV. The balance between Th1 and Th2 immunity was assessed based on the levels of antigen-specific serum IgG2a and IgG1 (Figure 4A). The result showed that among the adjuvants tested, alum, AddaVax, NanoSiO_2_, and Pam3CSK4 induced a significant increase in the expression of IgG1/IgG2a, which indicates that these adjuvants predominantly induce Th2 immunity when used with SV (Figure 4A). CSF1 protein expression is associated with M2 macrophage polarization (66, 67), which also involves characteristic inducing factors, receptor expression, and cytokine production for mediating Th2 responses (68). Certain reports revealed that a CSF1-dependent dendritic cell (DC) subset forms a link to Th2 immunity in lung immunity (69). An elevation in *Timp1* expression is considered to be induced in weakly injured lung tissues (70). Tissue injury or damage promotes the release of the damage-associated molecular patterns (DAMPs), some of which are known to act as potent inducers of Th2 immunity (71). Both *Timp1* and *Csf1* were considered biomarker genes in this study (Table 1). The ratio of *Csf1* plus *Timp1* expression levels to the sum of expression levels of all biomarker genes in total biomarker gene expression levels was indicated in Figure 4B. To investigate whether the ratio of *Csf1* plus *Timp1* expression levels to the sum of expression levels of all biomarker genes is an indicator of Th2 immunity, the tested adjuvants were divided into two groups based on the following criteria: IgG1/IgG2a ratio >10 or ≤ 10 (Figure 4C). The result indicated that the ratio of *Timp1* plus *Csf1* expression was significantly higher in the “IgG1/IgG2a >10” group compared to that in the “IgG1/IgG2a ≤ 10” group (Figure 4C). However, the changes in BALF IgA levels did not correspond significantly to the ratio of *Csf1* plus *Timp1* expression levels to the sum of expression levels of all biomarker genes between the two groups (Figure 4C). These results suggest that the ratio of *Csf1* plus *Timp1* expression levels to the sum of expression levels of all biomarker genes might be useful for predicting adjuvant-induced Th2 immunity.

### *Timp1* as a Marker for Cytotoxicity and BALF IgA Expression

**Figure 5:**
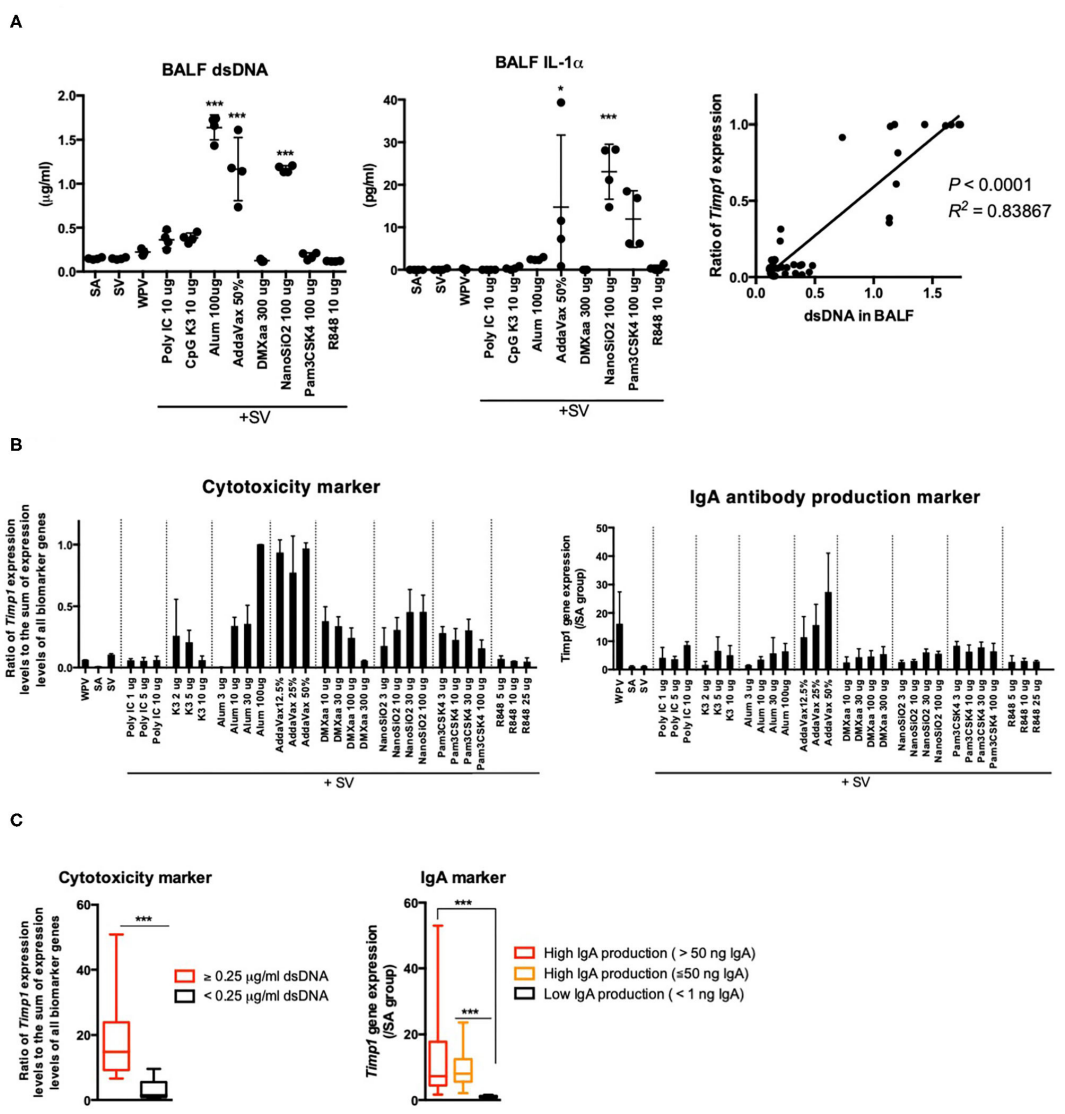
The elevation of *Timp1* expression levels reflects BALF IgA expression and cytotoxicity induced in response to nasal vaccination. **(A)** BALF dsDNA and IL-1α concentrations were measured 16 h after nasal inoculation. In addition, the lung biomarker gene expression levels were also analyzed. The ratio of *Timp1* expression levels to the sum of expression levels of all biomarker genes in total biomarker gene expression levels were calculated. The correlation between the ratio of *Timp1* expression levels to the sum of expression levels of all biomarker genes and dsDNA concentration in BALF were represented in a graph. **(B)** *Timp1* expression levels and ratio of *Timp1* expression levels at 16 h after nasal inoculation. **(C)** Changes in ratio of *Timp1* expression levels to the sum of expression levels of all biomarker genes and *Timp1* expression levels induced by the tested adjuvants are indicated individually by according to the BALF dsDNA concentration or antigen-specific IgA production induced in lungs. Data are represented in box-and-whisker plots, which indicate the median value (black bar inside box), the 25th and 75th percentiles (bottom and top of box, respectively), and minimum and maximum values (bottom and top whisker, respectively) **(C)**. Other results are expressed in terms of mean ± SEM [*n* = 4 for **(A,B)**, or 13–38 for **(C)** in each group]. *p < 0.05 and ***p < 0.001 compared with SA **(A)** (Dunnett's multiple comparison test). ***p < 0.01 **(C)** (Student's *t*-test).

Certain adjuvants elicit weak cytotoxicity, which is critical for improving vaccine efficacy. Alum and AddaVax elicit weak cytotoxicity, which involves the release of DAMPs from injured cells (or tissues) (58–60). The release of DAMPs, such as dsDNA (58), IL-33 (72), high mobility group box-1 (HMGB-1) (73), and IL-1α (74), has been reported to be associated with adjuvant efficacy. Among these, IL-33 (72, 74) and IL-1α (74) are reported to serve as critical mediators of IgA expression in response to nasal vaccination. To evaluate the cytotoxicity of the tested adjuvants, the concentration of dsDNA [the host DNA, an indicator of cell death (45)] in BALF specimens was measured 16 h after nasal vaccination (Figure 5A). The result indicated the concentration of dsDNA in BALF was significantly elevated upon treatment with adjuvants that induce the release of DAMPs (58–61) (Figure 5A). To investigate whether the release of dsDNA was associated with the release of DAMPs, the concentrations of IL-1α and IL-33 in BALF samples were measured. The levels of IL-1α 16 h after inoculation were notably elevated in mice treated with AddaVax, NanoSiO_2_, and Pam3CSK4, and marginally elevated in those treated with alum (Figure 5A), which has been reported to elicit weak cytotoxicity (58–61). IL-33 was not detected in BALF collected from any of the adjuvant-inoculated mice (data not shown). The ratio of *Timp1* expression levels to the sum of expression levels of all biomarker genes tended to correlate with the levels of dsDNA in BALF (Figure 5A). The *Timp1* expression levels and the ratio of *Timp1* expression levels to the sum of expression levels of all biomarker genes were notably elevated in mice that were treated with adjuvants that elicited cytotoxicity (Figure 5B). These results suggest that elevation in *Timp1* expression is a useful predictor of cytotoxicity and is associated with the release of DAMPs in lungs. To assess the potential of *Timp1* as an adjuvant efficacy marker, *Timp1* expression patterns were assessed by dividing the mice into three groups based on the following criteria: high IgA production (> 50 ng/mL IgA), high IgA production (≤ 50 ng/mL IgA), and low IgA production (<1 ng/ml IgA). Compared to the low IgA production group, the high IgA production groups exhibited a significant increase in the expression levels of *Timp1* upon treatment with adjuvants (Figure 5C). Furthermore, the high dsDNA levels compared to that in the SA group indicated a significant elevation in the ratio of *Timp1* expression levels to the sum of expression levels of all biomarker genes (Figure 5C). Based on these, we concluded that changes in *Timp1* expression in lung tissues 16 h after priming may be useful for predicting DAMP-mediated IgA production and cytotoxicity of nasal vaccine.

### Analysis of Genes Associated With Antigen Processing for Prediction of Adjuvant-Induced CTL Activities

**Figure 6:**
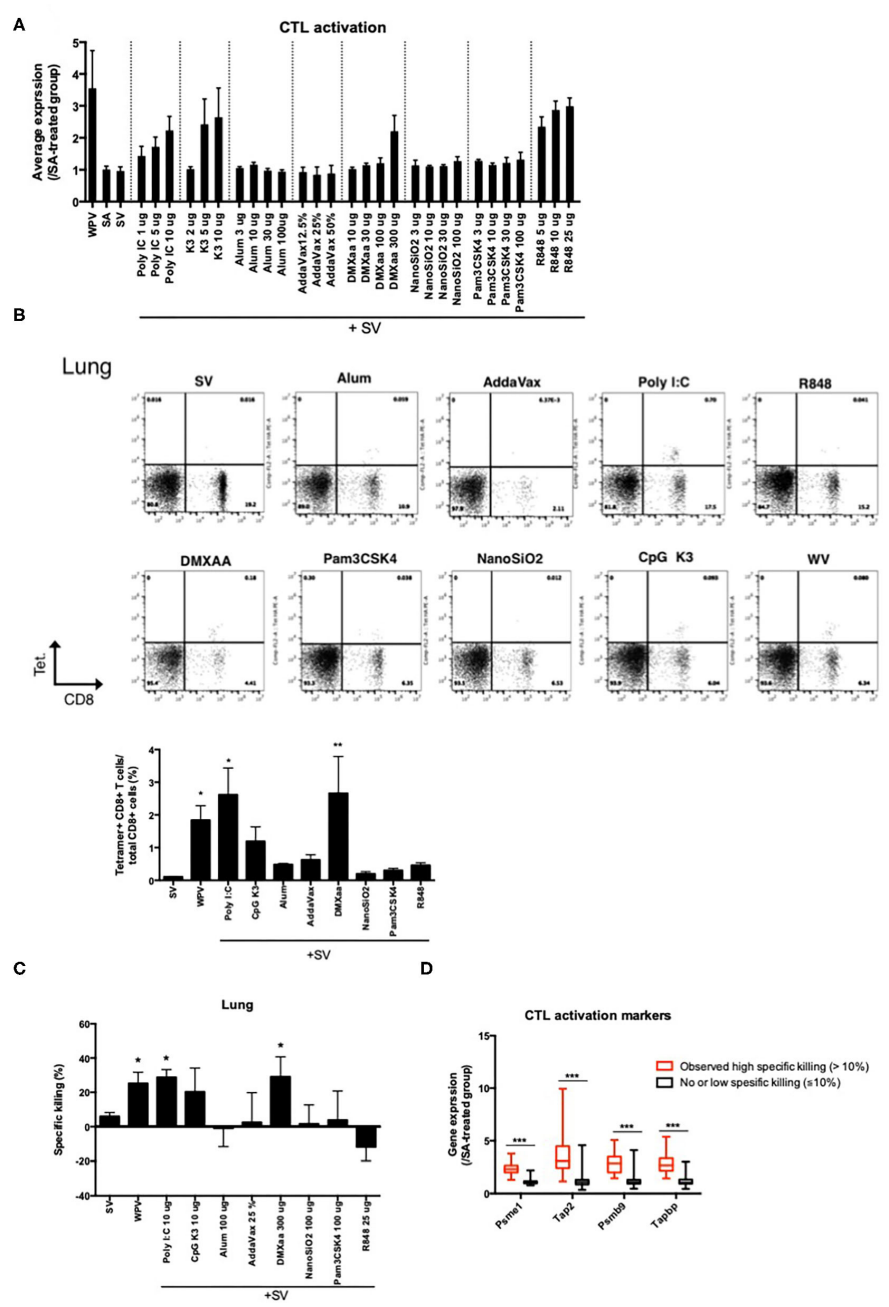
The elevation of proteasome-related gene expression levels reflects CTL activities in lungs induced in response to vaccination. **(A)** Average levels of proteasome-related gene expression in lung 14 days after final vaccination. Binding of CD8^+^ T cells in lung after two-time vaccination with H2-K^d^ bearing influenza HA peptide IYSTVASSL tetramer was assessed using FACS **(B)**. Specific killing of vaccine antigen presenting cells in immunized mice was assessed in the *in vivo* killing assay **(C)**. The changes in the expression levels of the four genes in response to adjuvant treatment are individually indicated according to the specific killing levels **(D)**. Data are represented in box-and-whisker plots, which indicate the median value (black bar inside box), the 25th and 75th percentiles (bottom and top of box, respectively), and minimum and maximum values (bottom and top whisker, respectively) **(D)**. Other results are expressed in terms of mean ± SEM [*n* = 4 for **(A–C)** or 18–50 for **(D)** in each group]. *p < 0.05 and **p < 0.01 compared with SV **(B,C)** (Dunnett's multiple comparison test). ***p < 0.001 **(D)** (Student's *t*-test).

**Figure 7:**
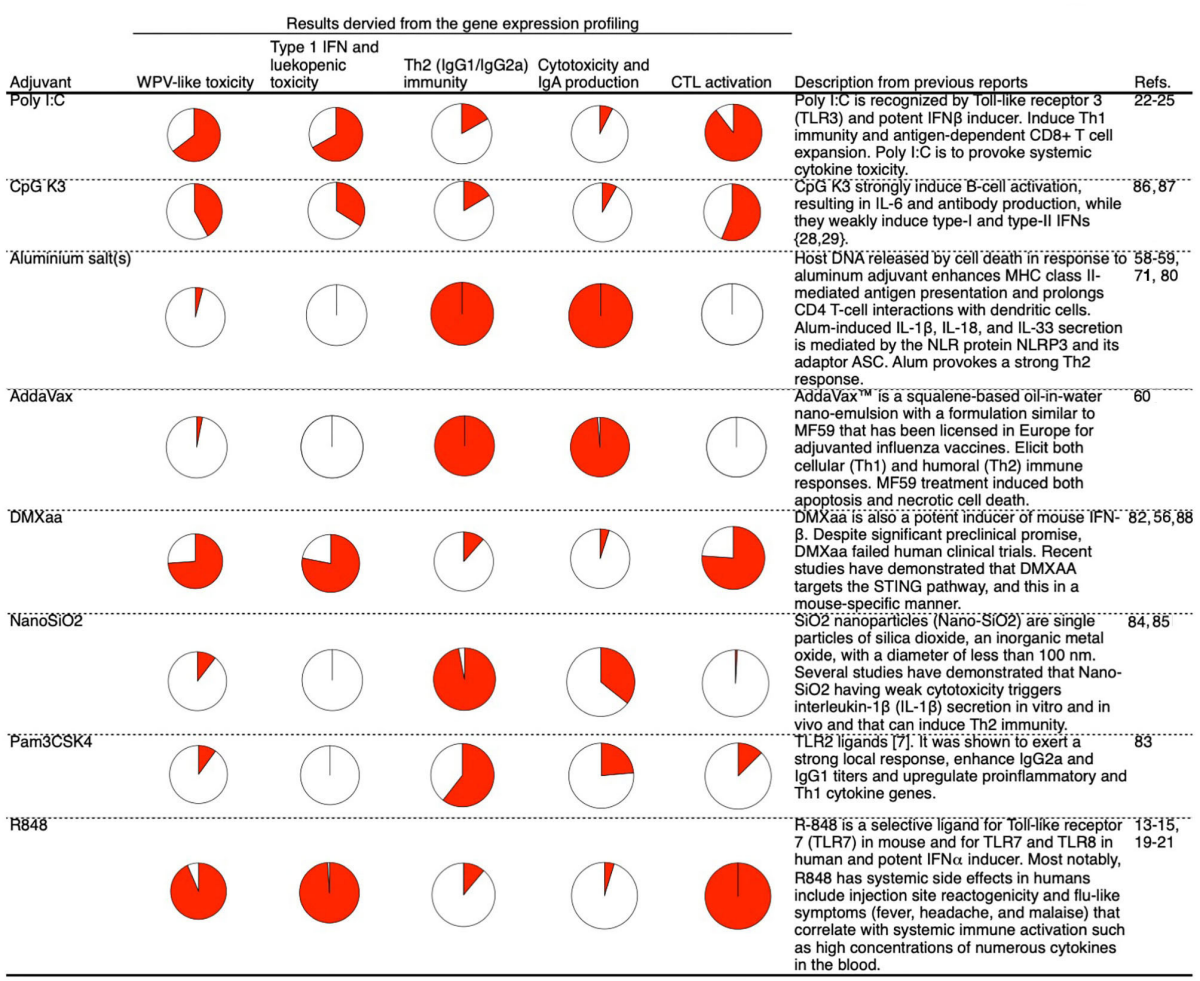
Summary of the mode of action profiling of tested adjuvants based on the biomarker gene expression profiles in lung 16 h after priming. Each activity is represented by a pie chart in which the area indicated by solid red represents the level of activity. The presented levels of activities were measured at a point at which WPV activity was maximum.

Lastly, we attempted to identify the genes associated with antigen presentation by major histocompatibility complex (MHC) class 1 for CTL activation, as antigen-specific CTLs are critical for preventing the expansion of infected cells. Among the biomarker genes, the following genes related to the transporter associated with antigen processing or the subunit of proteasome were evaluated: *Psme1, Tap2, Psmb9*, and *Tapbp* (Supplementary Figure 1). The average expression levels of these genes are indicated in Figure 6A. Treatment with type 1 IFN-inducing adjuvants and WPV was associated with relatively high expression levels (Figure 6A). To investigate whether the number of CTLs that express T cell receptor (TCR) for recognition of vaccine antigens are increased in response to adjuvant treatment, the binding of CD8^+^ T cells in lungs to the H2-K^d^ bearing influenza HA peptide IYSTVASSL tetramer after two rounds of vaccinations was assessed using fluorescence-activated cell sorting (FACS) analyses. The results indicated that except mice that were treated with R848, those treated with other type 1 IFN-inducing adjuvants exhibited tetramer binding by CD8^+^ T cells in lungs (Figure 6B). The concentration of tetramer binding CD8^+^ cells increased significantly in mice treated with WPV or poly I:C- or DMXaa-adjuvanted SV compared to those treated with SV (Figure 6B). Although the number of tetramer binding CD8^+^ cells increased in mice treated with CpG K3-adjuvanted SV, it did not differ significantly from that in the SV group. Furthermore, to directly assess vaccine-induced CTL activities *in vivo*, an *in vivo* CTL killing assay was performed. The results indicated that vaccine antigen-specific killing increased significantly in mice treated with WPV, poly I:C-, and DMXaa-adjuvanted SV compared to that in mice treated only with SV (Figure 6C). Although specific killing increased in the CpG K3-adjuvanted SV group, it did not differ significantly from that observed in the SV group. These results indicate that apart from R848, type 1 IFN-inducing adjuvants activate CTLs that express vaccine antigen-specific TCR. The tested adjuvants were divided into two groups: those that significantly increased tetramer binding by CD8^+^ T cells and induced higher specific killing compared when adjuvanted with SV or those that did not. Next, the expression levels of the four genes were assessed in each group. The result showed that the expression levels of all four genes were significantly elevated in the group treated with adjuvants that could induce CTL activation (Figure 6C). Based on these results, the expression levels of *Psme1, Psmb9, Tap2*, and *Tapbp* 16 h after priming were determined to be useful predictors of CTL activation. The profiling of commercially available adjuvants is summarized in Figure 7. We concluded that biomarker gene expression profiles in lungs at 16 h after priming can provide abundant information related to adjuvanted vaccine-induced protective immunity (vaccine efficacy) and toxicity.

## Discussion

Adjuvants are used with inactivated vaccines to enhance the immune response to antigens and the strength and persistence of the resulting immunity. The genomic profiling of immune responses induced by vaccine adjuvants improves our understanding of the mechanism of action of adjuvants. This can guide the rational design of vaccination strategies. Earlier studies have adopted systems biology approaches for profiling the priming properties of various vaccine adjuvants in preclinical models using genome-wide microarrays (75), as well as to analyze the responses in humans to adjuvanted and non-adjuvanted influenza vaccines (30, 31).

A clinical study conducted on infants vaccinated with trivalent SV with/without the MF59 adjuvant revealed that the inclusion of an oil-in-water adjuvant resulted in more rapid post-vaccination responses, including a greater interferon response (34), and also resulted in a stronger transcriptional response at certain time points and a higher hemagglutination inhibition titer (34). However, no method had been developed previously for rapid adjuvant profiling, in which genomic analysis can be used to assess the efficacy and toxicity of adjuvanted vaccines. Additionally, the process by which early post-priming genomic analysis data can provide information regarding vaccine efficacy and toxicity is poorly understood.

In this study, we investigated whether the biomarker gene expression profile in lungs at 16 h post-priming can be used to predict the efficacy (IgA antibody production, Th1/Th2 immune balance, and CTL activation) and toxicity (leukopenic toxicity and cytotoxicity) of adjuvanted/non-adjuvanted vaccines.

The biomarker genes selected were those associated with TLR signaling, IFN-mediated signaling, and antigen processing (Tables 1,3). Generally, TLR signals and type 1 and type 2 IFN can accelerate Th1 immunity (76, 77). With respect to the type of immunity, the expression patterns of most biomarker genes primarily indicate that Th1 immunity was induced. This study showed that the elevation in the average expression levels of biomarker genes was associated with the productivity of IgG2a antibody in mice (Figure 1C). Excessive IFN production can lead to the induction of toxicity. Earlier, WPV (15), poly I:C (22–25), and R848 (19–21), which induce potent type 1 IFN expression, were also observed to cause cytokine-mediated side effects in humans. In animal experiments, the loss of body weight and type 1 IFN-mediated leukopenic toxicity were observed upon treatment with WPV and poly I:C (38, 65). Therefore, the ability to induce type 1 IFN-mediated signals should be assessed with respect to toxicity. Among the biomarker genes, the three genes could predict WPV- or adjuvant-induced leukopenic toxicity with the highest accuracy (Figure 3D).

The biomarker genes were roughly divided into those that predicted the efficacy of type 1 IFN-inducing and non-type 1 IFN-inducing adjuvants (Figure 2A). However, certain genes such as *Timp1* and *Cxcl11* followed a unique expression pattern (Figure 2A). These two genes contribute strongly to the classification of Pam3CSK4, a type 2 IFN (IFNγ)-inducing adjuvant (62). Furthermore, functionally, the two genes were predicted to be involved in IFNγ signaling (Figure 2B). These results prompted us to hypothesize that individual consideration of the functions of each gene may provide detailed information on the toxicity and efficacy of adjuvants.

In this study, we first assessed adjuvant efficacy and the toxicity evaluation potential of biomarker genes based on gene function. CSF1 protein is associated with M2 macrophage polarization (66, 67), and M2 macrophages have characteristic inducing factors, receptor expression, and cytokine production to mediate Th2 responses (68). Certain reports showed that CSF1-dependent DC subsets form a link between Th2 immunity and lung immunity (69). TIMP1 protein, a secretory protein that inhibits the action of matrix metalloproteinases (MMPs), has been found to be associated with lung inflammation. *Timp1* expression increases following bleomycin injury and is localized to the inflammatory foci of the injured lung, which suggests its role in inflammation regulation (70). As reported, *Timp1* gene induction was spatially restricted to areas of lung injury (70), which suggests that *Timp1* expression in lungs can be activated upon lung tissue injury. Tissue injury or damage promotes the release of the DAMPs, which are known to act as potent Th2 inducers (71). Alum-induced IL-1α and HMGB1 are well-established DAMP molecules known to induce DC-dependent Th2 polarization (78). In the present study, the ratio of *Csf1* plus *Timp1* expression levels to the sum of expression levels of all biomarker genes was observed to be high in mice treated with in Th2 immunity-inducing adjuvants (Figure 4C). Furthermore, elevation of the r ratio of *Timp1* expression levels to the sum of expression levels of all biomarker genes was observed in mice treated with alum, AddaVax, and NanoSiO_2_, which are commonly known to induce the release of DAMPs or elicit cytotoxicity (58–61) (Figure 5B). The ratio of *Timp1* expression levels to the sum of expression levels of all biomarker genes and the BALF dsDNA concentrations tended to correlate (Figures 5A,B). In lungs, the BALF dsDNA concentrations and the IL-1α levels tended to correlate, which suggests that *Timp1* acts as a marker for cytotoxicity in lungs and is associated with the release of DAMPs (Figures 5A,B). DAMPs are important for exerting adjuvant efficacy as they accelerate innate immunity by enhancing mucosal IgA expression (72, 74). The *Timp1* expression levels were found to have excellent potential as predictors of BALF IgA production in nasal influenza vaccine (Figure 5C). Apart from DAMPs-inducing adjuvants, type 1 IFN-inducing adjuvants also induced an elevation in *Timp1* expression (Figure 5B). The results indicate that TLR ligands alter *Timp1* expression (79), which suggests that TLR-mediated signals might also contribute to the elevation of *Timp1* expression by certain adjuvants.

The four genes *Psme1, Psmb9, Tap2*, and *Tapbp*, which were selected as the biomarker genes in this study (Table 1), are associated with antigen presentation via MHC class 1 (Supplementary Figure 1). The expression of these genes is increased significantly upon treatment with WPV with CTL activation ability (Figure 6A). A higher expression of these genes might be indicative of cross presentation by DCs. This study shows that the binding of a tetramer with vaccine antigen-specific peptide to CD8^+^ cells was observed in mice treated with WPV and poly I:C-, CpG K3-, and DMXaa-adjuvanted SV (Figure 6B). In addition, among the tested adjuvants, these adjuvants in conjugation with the vaccines have a relatively high ability to induce specific killing using vaccine antigen-presenting cells (Figure 6C). The expression levels of *Psme1, Psmb9, Tap2*, and *Tapbp* were significantly elevated in mice treated with adjuvants that exhibit highly specific killing (Figure 6D), which suggests that these genes may contribute to cross presentation of the vaccine antigen and the processes may be accelerated by certain adjuvants.

This study revealed that the efficacy and toxicity of nasal influenza vaccine can be evaluated by observing the biomarker gene expression profile in the lung 16 h after priming. Intranasal vaccines can induce IgA production in the lungs and nasal cavity. From the viewpoint of evaluating the immunogenicity elicited by a vaccine, this method is not limited to nasal vaccines and may be applicable to the evaluation of immunogenicity of subcutaneous vaccines as well. Conversely, it is known that the DAMPs released by the vaccine adjuvants vary depending on the inoculation site (45, 80). DAMPs are known to accelerate immunity related to the efficacy of nasal vaccination as well as subcutaneous or intramuscular vaccination (58–60). Furthermore, since systemic immunization cannot lead to the induction of mucosal IgA expression, which can be achieved through nasal immunization, there is a difference in the mechanism underlying the immune response induced depending on the inoculation route (81). Therefore, the method proposed in this study cannot be considered adequate for confirming the efficacy of a reaction specific to the inoculation route in vaccines other than intranasal vaccination.

As shown in Figure 7, the presented method helps acquire information about various adjuvants. The majority of gene expression analyses that used these biomarkers were qualitative analyses, as quantitative analyses are considered challenging. Lung tissues were collected after 16 h of priming and used as specimens for the analysis of biomarker gene expression. Based on this, it is not possible to simultaneously analyze gene expression in the lungs along with IgG and IgA expression and CTL activity (excluding the verification of acute phenotypes such as cytotoxicity and leukopenic toxicity). Therefore, it was not possible to quantitatively analyze the correlation between the biomarker gene expression levels and vaccine efficacy. To perform a quantitative analysis, it is necessary to employ samples such as blood specimens that can be analyzed in a time-dependent manner. We expect that the biomarker gene-based analysis can be extended to samples other than lung tissues in future.

In this study, we first demonstrated that the efficacy and toxicity of adjuvants could be evaluated based on the biomarker gene expression profiles at 16 h after priming. This method may be effectively used for screening adjuvants and acquiring information in preclinical studies. Since screening at an early stage can be performed using samples collected within 24 h after priming, the method is suitable for screening within a short period. In other words, while screening is useful for narrowing down on the optimal adjuvant, studies on detailed immune profiling (such as studies on the effects on boosting) must be performed individually. Based on this, we believe that from a screening viewpoint, performing the analysis during priming, such as searching for compounds with adjuvant activity from compound libraries, is useful. We also believe that the screening results can provide information necessary for the selection of an optimal and more suitable adjuvant. Improving the effectiveness and safety of vaccines is important for protecting individuals from infectious diseases. Genomic assays are expected to serve as important tools in future as they enhance the safety and efficacy and demonstrate the suitability of various vaccine adjuvant formulations.

## Data Availability Statement

All datasets generated for this study are included in the article/Supplementary Material.

## Ethics Statement

The animal study was reviewed and approved by Animal Care and Use Committee of the National Institute of Infectious Diseases.

## Author Contributions

ES designed the study, interpreted the data, and wrote the manuscript. ES, HA, HM, TM, and KF performed the experiments. ES, TM, and IH proofread and corrected the final manuscript. The authors approved the final version.

## Conflict of Interest

The authors declare that the research was conducted in the absence of any commercial or financial relationships that could be construed as a potential conflict of interest.
