# Supplementary figures and images for: Immunogenicity and Toxicity of Different Adjuvants Can Be Characterized by Profiling Lung Biomarker Genes After Nasal Immunization
Source: Front Immunol. 2020 Sep 11;11:2171. doi: 10.3389/fimmu.2020.02171 (PMC7516075; doi:10.3389/fimmu.2020.02171)

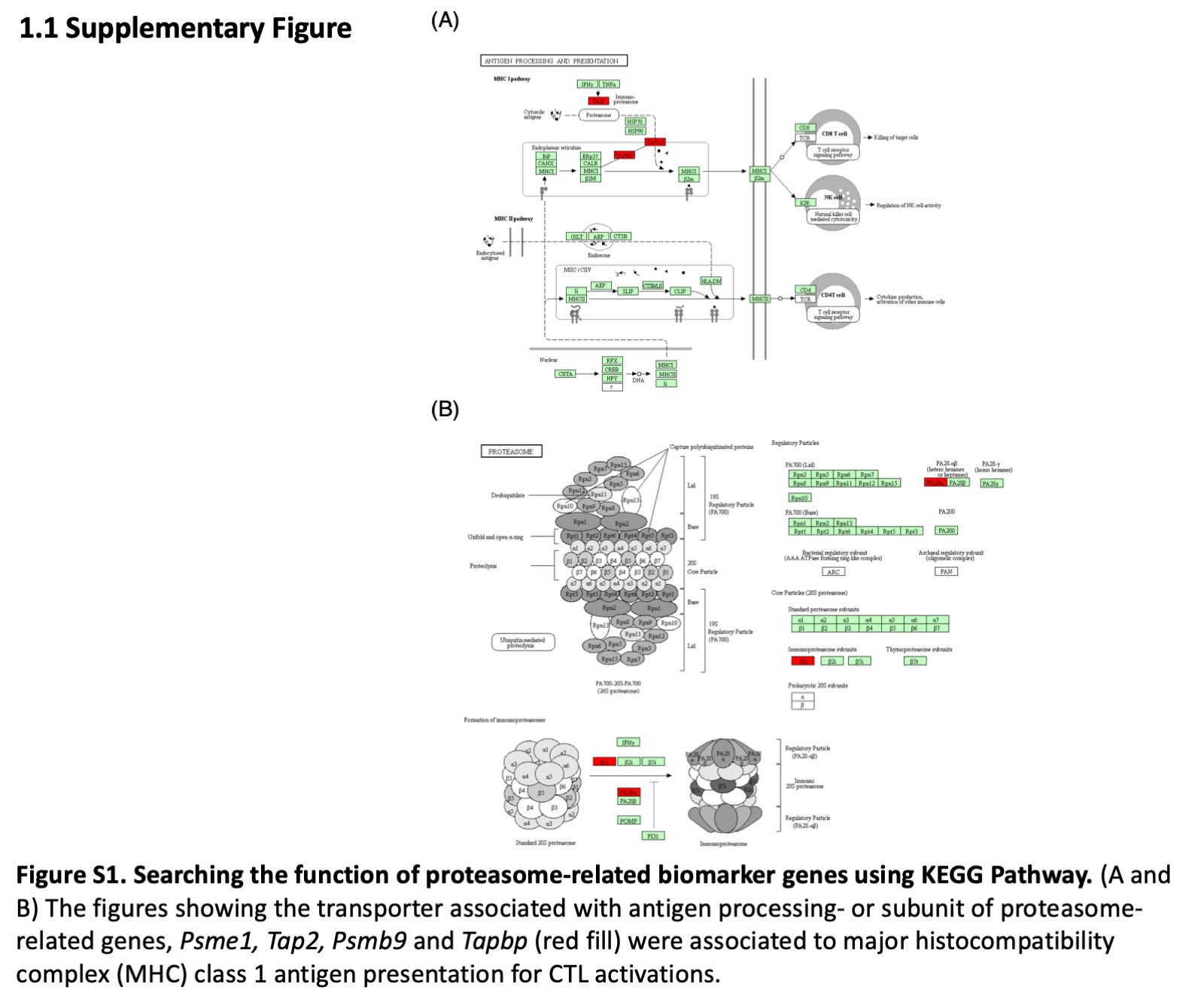

Supplement: Supplementary file 1 [file Image_1.JPEG]
