# Supplementary material for: Immunogenicity and Toxicity of Different Adjuvants Can Be Characterized by Profiling Lung Biomarker Genes After Nasal Immunization
Source: Front Immunol. 2020 Sep 11;11:2171. doi: 10.3389/fimmu.2020.02171 (PMC7516075; doi:10.3389/fimmu.2020.02171)
Supplement: Supplementary file 2 [file Table_1.DOCX]

***Supplementary Material***

**Supplemental table 1. The biomarker genes expression levels in lung**
